# Breaking the p-type doping barrier in β-Ga_2_O_3_: a GaN-based heterojunction bipolar transistor with high gain, high breakdown, and RF capability

**DOI:** 10.1039/d5ra07197f

**Published:** 2025-10-08

**Authors:** Phuc Hong Than, Tho Quang Than, Yasushi Takaki

**Affiliations:** a Duy Tan University (DTU) 3 Quang Trung, Hai Chau Danang 550000 Vietnam thanhongphuc@duytan.edu.vn; b Central Power Corporation (EVNCPC) 78A Duy Tan, Hoa Thuan Dong, Hai Chau Danang 550000 Vietnam; c Power Device Works, Mitsubishi Electric Corporation 997, Miyoshi Koushi-shi Kumamoto 861-1197 Japan

## Abstract

Despite extensive research on unipolar β-Ga_2_O_3_ semiconductor devices, the advancement of bipolar devices, particularly heterojunction bipolar transistors (HBTs), has been significantly hindered by the lack of reliable p-type doping in β-Ga_2_O_3_. In this paper, we present the first comprehensive simulation study of a functional HBT based on an n-type β-Ga_2_O_3_ emitter, a p-type GaN base, and an n-type GaN collector, aiming to address the critical challenge of p-type doping in β-Ga_2_O_3_ for bipolar devices. The proposed Ga_2_O_3_/GaN HBT, simulated with full consideration of traps, exhibits a maximum DC current gain (*β*_DC_) of 18.3, a high collector current density (*J*_C_) of 14.3 kA cm^−2^, a collector–base breakdown voltage (BV_CBO_) of 120 V, a power figure of merit (PFOM) of 41.3 MW cm^−2^, and a low specific on-resistance (*R*_on,sp_) of 0.35 mΩ cm^2^. The temperature-dependent current–voltage (*I*–*V*) characteristics from 300 K to 460 K reveal stable operation up to 460 K, albeit with a 31.1% reduction in *β*_DC_ and a 30.0% decline in PFOM due to carrier mobility degradation and enhanced recombination. Furthermore, device performance was optimized by engineering the base and collector thicknesses. The results indicate that a thin base (0.05 μm) maximizes *β*_DC_, while a thick collector (2.0 μm) boosts PFOM to 138 MW cm^−2^ without compromising gain. In addition, high-frequency simulations show a cutoff frequency (*f*_T_) of 30 GHz at 300 K, confirming the device's suitability for RF and power-switching applications. These results indicate that the Ga_2_O_3_/GaN HBT is a promising candidate for next-generation power electronics, owing to its unique combination of high breakdown voltage and excellent frequency performance.

## Abstract 2

Despite extensive research on unipolar β-Ga_2_O_3_ semiconductor devices, the advancement of bipolar devices, particularly heterojunction bipolar transistors (HBTs), has been significantly hindered by the lack of reliable p-type doping in β-Ga_2_O_3_.

## Graphical abstract

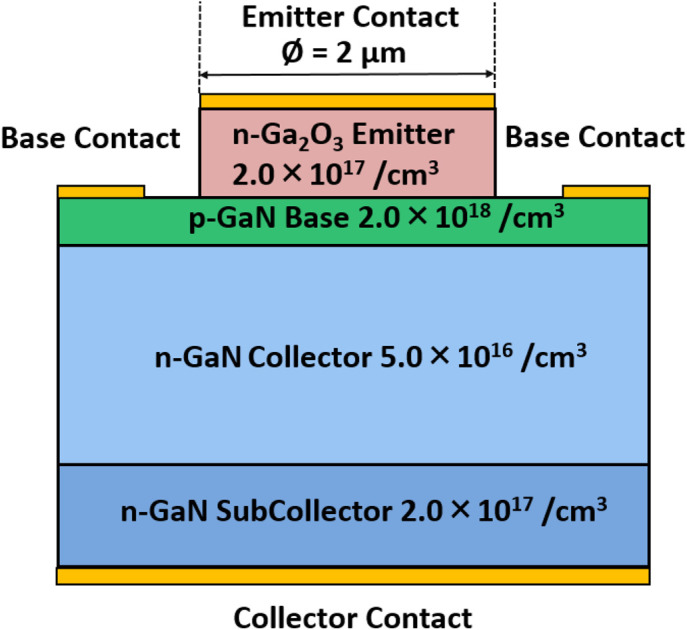

## 1 Introduction

Among wide-bandgap semiconductors such as silicon carbide (SiC), gallium nitride (GaN), and gallium oxide (Ga_2_O_3_), beta-gallium oxide (β-Ga_2_O_3_) has garnered significant attention due to its ultra-wide bandgap of ∼4.8 eV, high breakdown electric field exceeding 8 MV cm^−1^, excellent controllability of n-type conductivity through intentional donor doping over a broad carrier concentration range (10^15^–10^20^ cm^−3^), and the availability of large-area, low-defect single-crystal substrates grown by melt-based techniques, which are compatible with various epitaxial growth methods.^1–6^ Thus, over the past decade, extensive research and development has focused on unipolar devices based on β-Ga_2_O_3_ for next-generation high-power and high-frequency applications, such as Schottky barrier diodes (SBDs), metal-oxide-semiconductor field-effect transistors (MOSFETs), and high-electron-mobility transistors (HEMTs).^7–20^ Prior research at the National Institute of Information and Communications Technology (NICT, Tokyo, Japan), where one of the authors participated, showed that normally-off lateral β-Ga_2_O_3_ MOSFETs with nitrogen-doped channels were feasible. The use of nitrogen as a deep acceptor for current-blocking layers in β-Ga_2_O_3_-based power devices was validated by the stable performance of these devices at a threshold voltage of +5 V.^21,22^ However, the lack of reliable p-type doping in β-Ga_2_O_3_ has significantly limited the development and investigation of bipolar devices based on this material. As a key technology in high-speed electronics, heterojunction bipolar transistors (HBTs) offer superior frequency response and power-handling capabilities compared to other bipolar devices, making them essential for next-generation RF and power-switching applications. Recently, M. Mehta *et al.* proposed the use of p-type oxides to realize HBTs based on β-Ga_2_O_3_.^23^ A β-Ga_2_O_3_ HBT employing a Cu_2_O base was simulated to estimate its power figure of merit (PFOM), but its performance was severely constrained by the low bandgap of Cu_2_O. To address this issue, the authors suggested using alternative p-type oxides with a bandgap of *E*_g_ > 3.4 eV and an electron diffusion length >0.4 μm to achieve PFOM values exceeding those of state-of-the-art β-Ga_2_O_3_ unipolar transistors. Promising candidates such as NiO and r-GeO_2_ were identified for their potential to significantly enhance PFOM.^23^ In this study, we propose a HBT structure based on n-type β-Ga_2_O_3_ and p-type GaN. GaN was chosen as the base material due to its wide bandgap of 3.53 eV, greater than the 3.4 eV threshold suggested by Mehta *et al.*, and its high compatibility with β-Ga_2_O_3_.^24,25^ Although Ga_2_O_3_/GaN heterostructures for various unipolar and optoelectronic applications have been widely studied and reported, including photodetectors,^26–28^ light-emitting diodes (LEDs),^29–31^ gate dielectrics or passivation layers in HEMTs/metal-oxide-semiconductor high-electron-mobility transistors (MOSHEMTs),^25,32–38^ MOSFETs,^39–43^ as well as p–n diodes,^44–47^ their potential in bipolar junction transistors remains entirely unexplored. To the best of our knowledge, no experimental or simulation study on a functional β-Ga_2_O_3_/GaN HBT has been reported to date. Here, we report the first comprehensive simulation-based demonstration of an n-β-Ga_2_O_3_/p-GaN/n-GaN HBT, incorporating realistic interfacial defects and compensation effects to provide a physically grounded evaluation of its feasibility, performance, thermal reliability, and optimization strategies under practical defect considerations.

## 2 Structure and mechanism

**Fig. 1:**
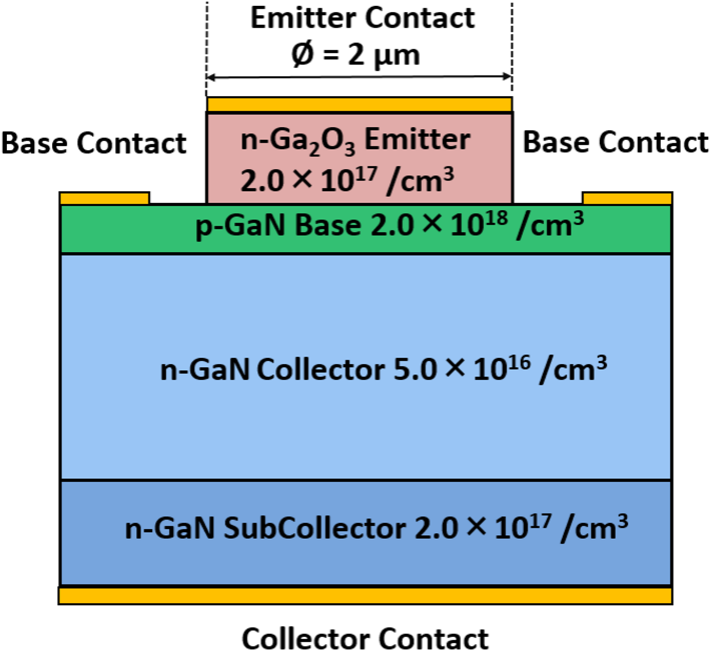
Cross-sectional schematic of the Ga_2_O_3_/GaN HBT. Only half of the structure was used for simulation due to symmetry, but the full structure is illustrated here.

The schematic of the simulated HBT structure consists of an n-β-Ga_2_O_3_ emitter layer with a thickness of 0.15 μm and a doping concentration of *N*_D_ = 2.0 × 10^17^ cm^−3^, a p-GaN base layer with a thickness of 0.05 μm and *N*_A_ = 2.0 × 10^18^ cm^−3^, an n-GaN collector layer with a thickness of 0.55 μm and *N*_D_ = 5.0 × 10^16^ cm^−3^, and an n-GaN subcollector layer with a thickness of 0.15 μm and *N*_D_ = 2.0 × 10^17^ cm^−3^, as illustrated in Fig. 1. Due to the lateral symmetry of the device structure, only half of the HBT was simulated in the TCAD environment with appropriate symmetry boundary conditions. The full cross-sectional structure is shown in Fig. 1 for clarity. The emitter contact is placed on top of the n-Ga_2_O_3_ emitter layer to form an ohmic contact, while the base contact is located on the p-GaN base layer. The collector contact is formed on the backside of the n-GaN subcollector layer, also establishing an ohmic contact. The structure, thicknesses, and doping concentrations of the emitter, base, and collector layers in the proposed HBT design are based on those used in InGaP/GaAs heterojunction phototransistors previously fabricated by our group, as reported in ref. 48–50. The key physical models and material parameters used in the simulation, as well as the calibration of model parameters using experimental data, have been described in detail elsewhere.^51,52^ In this study, a baseline HBT, referred to as the bulk-trap-only HBT (B-HBT), incorporating key defect-related effects such as deep-level electron traps in the Ga_2_O_3_ layer, was first established to enhance the accuracy of the simulations. These traps, located at *E*_C_ −0.6 eV, *E*_C_ −0.75 eV, and *E*_C_ −1.05 eV, correspond to the E1, E2, and E3 levels identified in bulk and epitaxial β-Ga_2_O_3_ films,^53–58^ and were included to model carrier trapping and recombination effects more accurately, thereby enhancing the realism of the simulation. To account for experimentally observed non-idealities, a full-trap HBT model, referred to as the F-HBT, was developed. In this case, to simulate the compensation effects induced by oxygen diffusion from the Ga_2_O_3_ into the p-GaN base, additional defect mechanisms were incorporated in the p-GaN layer: a shallow donor-like trap representing oxygen substituting nitrogen (O_N_, *E*_C_ −0.03 eV)^59–62^ and the dominant shallow acceptor (*E*_V_ +0.225 eV) commonly observed in GaN, which is enhanced by oxygen doping and is likely related to a V_Ga_–O–H complex.^63,64^ At the Ga_2_O_3_/GaN interface, an interface trap density (*D*_it_) was introduced to emulate interface defects. A value of *D*_it_ ≈ 1.0 × 10^12^ eV^−1^ cm^−2^ was used in our simulation. Although Qian Feng *et al.* reported even higher *D*_it_ values (2.3–5.3 × 10^13^ eV^−1^ cm^−2^) for mechanically exfoliated β-Ga_2_O_3_/GaN interfaces,^65^ using these values resulted in a complete loss of transistor action in our simulations. Therefore, a reduced *D*_it_ level was employed to ensure both numerical convergence and preservation of transistor behavior, while still reflecting the experimentally observed interfacial degradation. In addition, polarization-induced charges were enabled with a scaling factor to capture the effect of polarization mismatch and strain relaxation at the monoclinic β-Ga_2_O_3_/hexagonal GaN interface.^66^ This comprehensive trap and interface model (F-HBT) provides a physically grounded description of bulk and interfacial recombination pathways and allows a more rigorous assessment of the degradation mechanisms in Ga_2_O_3_/GaN HBTs. The modeling assumptions employed in the F-HBT model are not merely hypothetical but are substantiated by experimental findings reported in the literature.^66^ In particular, structural and chemical characterizations of β-Ga_2_O_3_/GaN heterostructures have consistently revealed interfacial traps, strain-induced dislocations, and oxygen-related defect formation, which provide the experimental basis for the adopted full-trap HBT model. Recent experimental characterizations have revealed that the β-Ga_2_O_3_/GaN heterointerface is far from ideal and strongly influences device performance. High-resolution TEM analyses provide clear evidence of non-abrupt junctions and interfacial defects. For example, Zhang *et al.*^67^ observed that β-Ga_2_O_3_ films grown on GaN substrates form a fuzzy transition layer of 3–4 atomic planes at the interface due to atomic reconfiguration, rather than a sharp boundary. Similarly, Seo *et al.*^68^ reported that grain boundaries and defect clusters originating from the Ga_2_O_3_/GaN junction propagate into the overgrown β-Ga_2_O_3_ layer, confirming the presence of interfacial defect networks. Structural characterizations further highlight the role of lattice mismatch and strain. XRD measurements indicate that GaN grown on β-Ga_2_O_3_ experiences a lattice mismatch of ∼4.7%, resulting in a threading dislocation density (TDD) of ∼4.0 × 10^7^ cm^−2^.^69^ Complementary Raman spectroscopy analyses demonstrated a red-shift of the *E*_2_ (high) phonon mode relative to bulk GaN, corresponding to compressive strain of 0.26 GPa in planar GaN/β-Ga_2_O_3_ LEDs and 0.07 GPa in nanorod structures.^31^ These results confirm that both dislocations and residual strain are inevitable at the heterointerface. Chemical analyses also point to interfacial reactions and defect formation. Pre-annealing of GaN substrates prior to β-Ga_2_O_3_ growth has been shown to substitute N atoms with O atoms, producing a GaN_*x*_O_*y*_ interfacial layer.^67^ This observation directly supports the formation of O_N_-related defects that compensate acceptors in the GaN base. In addition to ON-related compensation, the use of Mg-doped GaN substrates introduces a further risk associated with oxygen incorporation. Pankove *et al.*^70^ demonstrated that Mg and O co-doping leads to the formation of Mg–O complexes (‘molecular doping’), which effectively scavenge oxygen donors and render the material highly insulating. Such complexes would further reduce the effective hole concentration and thereby hinder hole injection at the Ga_2_O_3_/GaN interface. These characterizations consistently demonstrate that the β-Ga_2_O_3_/GaN heterointerface suffers from non-abrupt transitions, strain-induced dislocations, and oxygen-related defect formation.

## 3 Results and discussion

### 3.1 Electrical characteristics

**Fig. 2:**
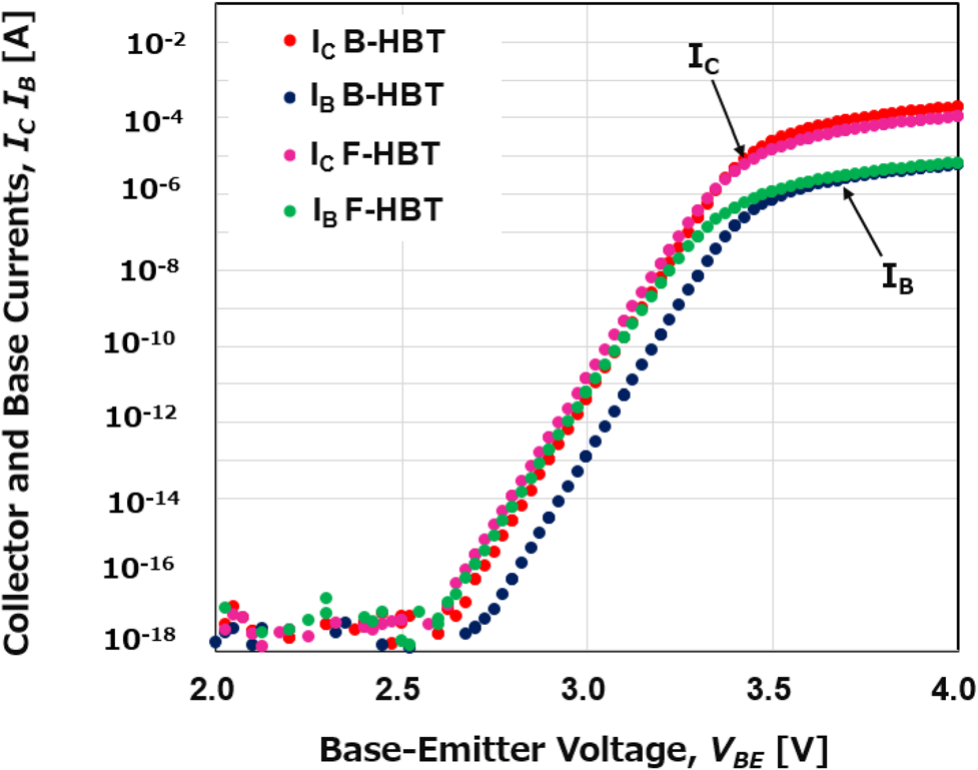
Room-temperature Gummel plots of the B-HBT and F-HBT with collector–base voltage *V*_CB_ = 0 V.

Fig. 2 presents the Gummel plots of the simulated Ga_2_O_3_/GaN B-HBT and F-HBT, showing the base current (*I*_B_) and collector current (*I*_C_) as functions of the base–emitter voltage (*V*_BE_) on a logarithmic scale, with the base–collector voltage (*V*_BC_) fixed at 0 V. A distinct onset of current amplification is observed when *V*_BE_ exceeds 2.7 V, marking the turn-on of transistor operation. This relatively high turn-on voltage is consistent with the wide bandgap nature of the n-Ga_2_O_3_/p-GaN heterojunction and validates the band alignment in the simulation. When bulk traps in Ga_2_O_3_ as well as comprehensive interfacial effects at the Ga_2_O_3_/GaN junction are included, the Gummel characteristics exhibit noticeable changes compared with the bulk-trap-only case. In particular, *I*_C_ decreases slightly while *I*_B_ increases, due to enhanced recombination and carrier capture introduced by defect-related mechanisms at the heterojunction (*e.g.*, interface states, atomic mixing, and non-ideal band profiles). Furthermore, oxygen diffusion from n-Ga_2_O_3_ emitter into the p-GaN base can generate compensating defects, lowering the effective hole concentration and injection efficiency. As a result, the DC current gain (*β*_DC_ = *I*_C_/*I*_B_) of the F-HBT remains in the approximate range of 12–18. This trend is physically reasonable and consistent with experimental reports that interfacial degradation strongly limits the gain of GaN-based HBTs.^71–73^ The exponential dependence of *I*_C_ and *I*_B_, the clear separation between collector and base currents, and the persistence of current amplification even in the presence of realistic defect modeling confirm that the proposed Ga_2_O_3_/GaN structure exhibits genuine heterojunction bipolar transistor behavior. Our simulations highlight both the intrinsic performance potential and the limitations imposed by non-ideal interfaces and traps, thereby validating the feasibility of the n-type β-Ga_2_O_3_ emitter/p-type GaN base/n-type GaN collector structure for future high-power HBT applications.

**Fig. 3:**
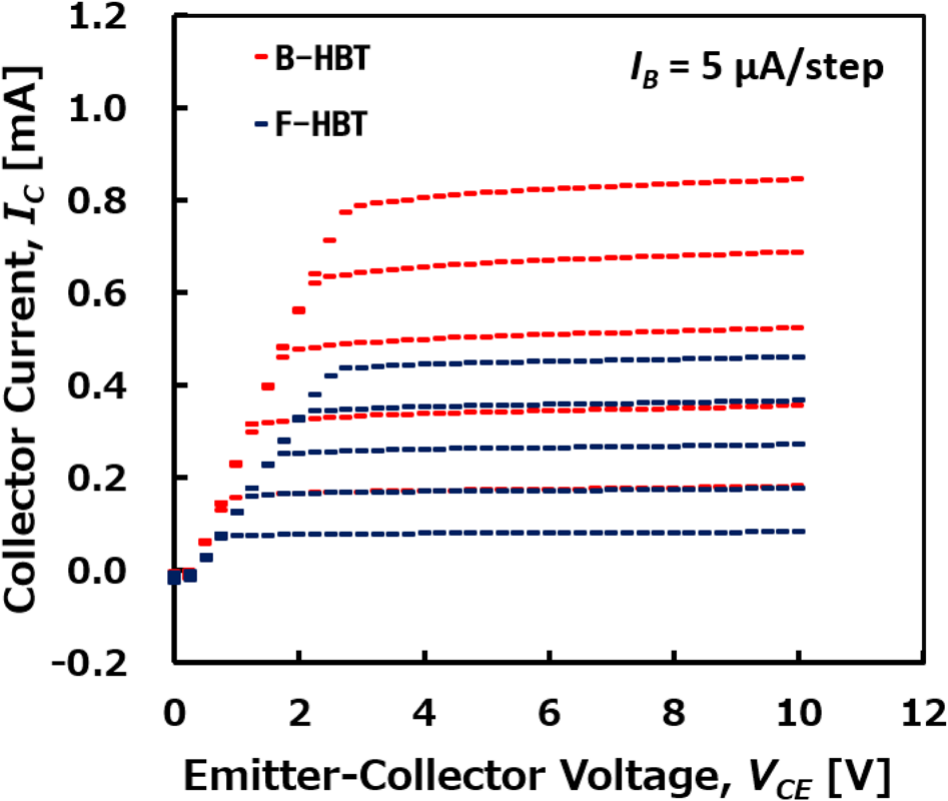
Room-temperature common-emitter *I*–*V* characteristics of the B-HBT and F-HBT for base currents varying from 5 μA to 25 μA in steps of 5 μA.

**Fig. 4:**
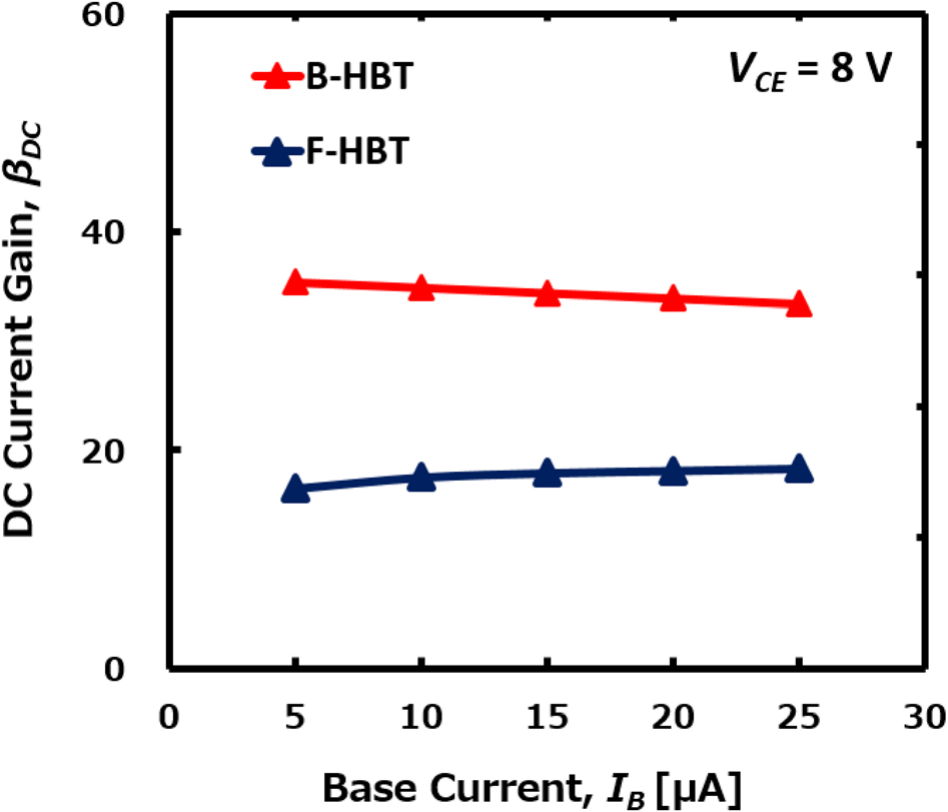
Room-temperature DC current gains of the B-HBT and F-HBT for various base currents.

The room-temperature common-emitter *I*–*V* characteristics of the simulated Ga_2_O_3_/GaN B-HBT and F-HBT are presented in Fig. 3. The base current (*I*_B_) was swept from 5 μA to 25 μA in steps of 5 μA, while the collector–emitter voltage (*V*_CE_) was varied from 0 to 10 V for each *I*_B_. An offset voltage (*V*_CE,offset_) as low as 0.25 V was observed, which is substantially lower than that of typical AlGaN/GaN HBTs (2 V)^73–75^ and comparable to InGaN/GaN HBTs (0.3 V).^76,77^ The DC current gain (*β*_DC_) was extracted from the output curves in Fig. 3 and plotted as a function of *I*_B_ in Fig. 4. With increasing *I*_B_, the collector current (*I*_C_) rises proportionally, indicating effective current modulation and forward-active transistor operation. Each output curve exhibits a steep increase in *I*_C_ at low *V*_CE_, followed by a well-defined saturation region where *I*_C_ remains nearly constant with further increases in *V*_CE_. This behavior reflects efficient carrier injection across the n-Ga_2_O_3_/p-GaN emitter–base junction and minimal base-width modulation (Early effect), highlighting excellent device stability and linearity—key requirements for high-frequency and high-power applications. Furthermore, as shown in Fig. 3, the collector current *I*_C_ of the F-HBT is lower than that of the B-HBT, and the F-HBT also exhibits a slight upward shift in the knee voltage. In addition, the saturation region of the F-HBT becomes flatter, indicating a smaller Early effect. The degradation in the F-HBT is attributed to additional recombination pathways introduced at the Ga_2_O_3_/GaN boundary, together with parasitic resistances and oxygen-related compensation in the p-GaN base. These trends are consistent with the Gummel plots shown in Fig. 2, further confirming the physical validity of the defect-inclusive model. Despite the incorporation of all relevant traps, the collector current *I*_C_ of the F-HBT still increases with *V*_CE_ and then saturates, confirming effective current modulation and forward-active operation similar to that of the B-HBT.

The specific on-resistance (*R*_on,sp_) of the B-HBT and F-HBT was determined to be 0.19 mΩ cm^2^ and 0.35 mΩ cm^2^, respectively, at *V*_CE_ = 5 V with *I*_B_ = 25 μA and *I*_C_ = 815 μA for the B-HBT and *I*_*C*_ = 450 μA for the F-HBT. These values were calculated based on a circular emitter region with a radius of 1.0 μm, corresponding to high collector current densities (*J*_C_) of 26 kA cm^−2^ and 14.3 kA cm^−2^, respectively. As shown in Fig. 4, the DC current gain of the B-HBT remains nearly constant or exhibits a slight decline as *I*_B_ increases, reflecting an almost idealized response dominated by bulk traps. In contrast, the F-HBT demonstrates a slight increase in *β*_DC_ with *I*_B_, which is consistent with our earlier experimental observations in InGaP/GaAs heterojunction phototransistors (HPTs).^48–50^ In those devices, *β*_DC_ also increased with *I*_B_ because recombination at the emitter perimeter and heterointerface became relatively less significant at higher injection levels, while the diffusion current dominated. A similar mechanism can be used to explain the behavior of the F-HBT: at low *I*_B_, interface and compensation defects dominate recombination, suppressing *β*_DC_, whereas at higher *I*_B_ these defects are partially saturated, allowing the diffusion current to prevail, thereby improving injection efficiency and slightly enhancing *β*_DC_. Specifically, *β*_DC_ in the B-HBT decreases modestly from 35.4 at *I*_B_ = 5 μA to 33.4 at *I*_B_ = 25 μA (5.7% reduction), whereas in the F-HBT it increases from 16.5 to 18.3 (11% enhancement) over the same bias range at *V*_CE_ = 8.0 V. This difference clearly demonstrates that the full-trap model more realistically captures the bias-dependent behavior observed in practical heterojunction devices.

As shown in Fig. 4, the DC current gain (*β*_DC_) of the F-HBT remains in the range of 16–18 for base currents (*I*_B_) between 5 and 25 μA, which is more than twice lower than that of the B-HBT. The reduced *β*_DC_ is associated with defect-assisted recombination at the Ga_2_O_3_/GaN boundary, originating from lattice mismatch, interdiffusion, and associated band-edge distortions. These defects act as strong recombination centers in the depletion region of the emitter–base junction, thereby increasing the base current and reducing the DC current gain. A similar mechanism was also observed in our previous studies on InGaP/GaAs HPTs, where defect generation at the heterointerface and at the emitter perimeter enhanced recombination and degraded both current gain and photoresponse. In the Ga_2_O_3_/GaN system, additional parasitic resistances at the heterointerface and compensating defects in the p-GaN base induced by oxygen diffusion further reduce the hole concentration and emitter injection efficiency. These findings are consistent with experimentally observed trends in GaN-based HBTs, providing a credible explanation for the performance limitations and highlighting that interface quality, defect passivation, and device design strategies are crucial to realize high-performance β-Ga_2_O_3_/GaN HBTs.

**Fig. 5:**
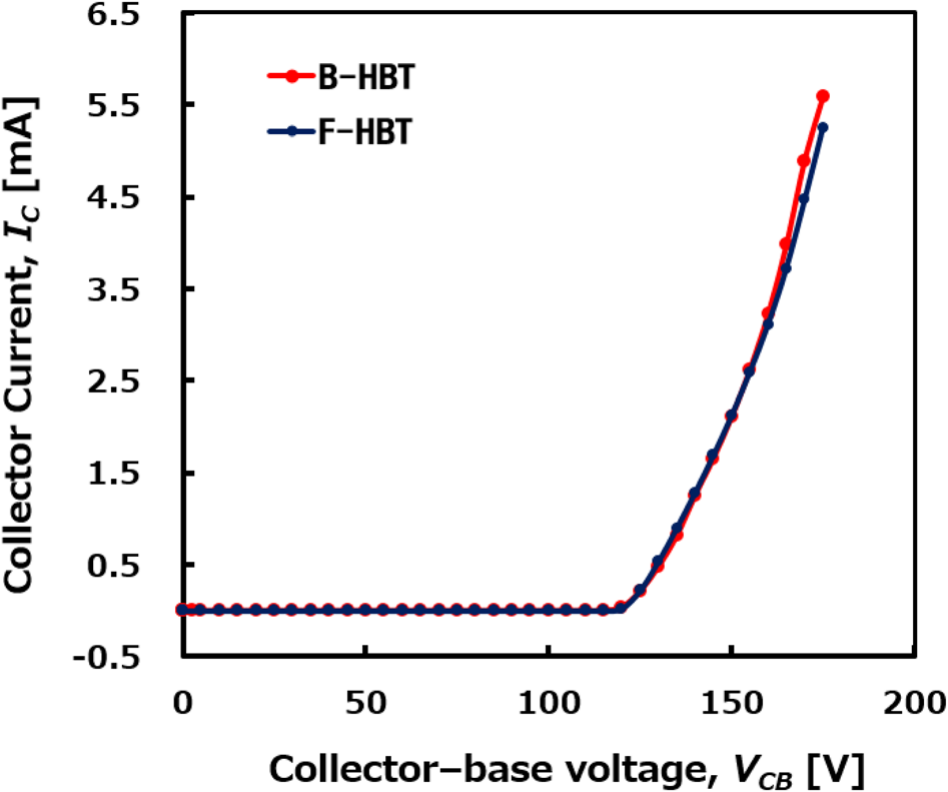
Simulated collector current as a function of the reverse-biased collector–base voltage (*V*_CB_) at room temperature, with both the base and emitter grounded. The sharp increase in *I*_C_ at approximately 120 V indicates the collector–base breakdown voltage (BV_CBO_) of the Ga_2_O_3_/GaN HBT.

The collector current (*I*_C_) as a function of the reverse-biased collector–base voltage (*V*_CB_), with both the base and emitter grounded, was simulated to determine the breakdown voltage of the HBT, as shown in Fig. 5. *V*_CB_ was swept from 0 V to 200 V to evaluate the collector–base breakdown behavior (BV_CBO_). For both the B-HBT and F-HBT, *I*_C_ remains nearly zero until a sharp increase occurs at approximately 120 V, which is identified as the collector–base breakdown voltage (BV_CBO_) of the Ga_2_O_3_/GaN HBT structure. However, the collector current of the F-HBT is slightly suppressed beyond breakdown compared to that of the B-HBT, reflecting the additional influence of interface-related defects and compensation effects. The transistor's power figure of merit (PFOM) for the B-HBT and F-HBT, defined as BV_CBO_^2^/*R*_on,sp_, was calculated to be 74.8 MW cm^−2^ and 41.3 MW cm^−2^, respectively. The high breakdown voltage, primarily governed by the intrinsic wide-bandgap nature of Ga_2_O_3_ and GaN together with the engineered heterojunction profile, demonstrates the robust reverse-blocking capability of the proposed HBT structure. Furthermore, the high PFOM confirms a favorable trade-off between breakdown performance and conduction loss, indicating strong potential for high-voltage power switching applications.

**Fig. 6:**
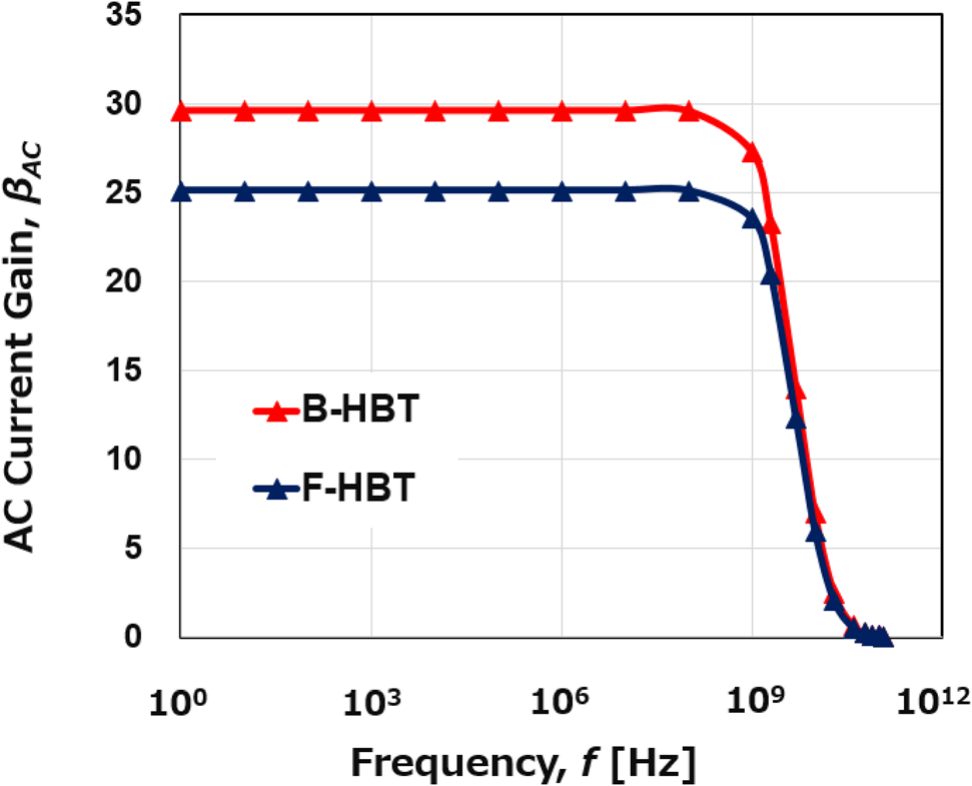
Simulated AC current gain (*β*_AC_) of the B-HBT and F-HBT as a function of frequency. The cutoff frequency (*f*_T_) is defined as the frequency at which the current gain drops to unity.

In addition to their application in high-voltage power switching, HBTs are also widely used in RF and high-speed switching circuits. Therefore, the high-frequency performance of the proposed Ga_2_O_3_/GaN HBT was evaluated by extracting the cutoff frequency (*f*_T_), defined as the frequency at which the short-circuit current gain drops to unity. As seen in Fig. 6, the AC current gain (*β*_AC_) remains nearly constant at 29.6 up to frequencies near 10^9^ Hz and then decreases rapidly, with *f*_T_ determined to be approximately 35 GHz for the B-HBT. By contrast, the F-HBT exhibits a lower low-frequency *β*_AC_ (25.1) compared to the B-HBT (29.6), with the roll-off occurring at a lower frequency. The cutoff frequency *f*_T_ of the F-HBT is reduced to approximately 30 GHz, which is 14.3% lower than that of the B-HBT. The reduction in *f*_T_ for the F-HBT is attributed to carrier recombination and trapping at the heterojunction, which increase the effective base transit time and parasitic resistances, thereby degrading the high-frequency response. Despite this reduction, both the B-HBT and F-HBT maintain effective operation at high frequencies. The simulated *f*_T_ values are consistent with the device's structural parameters, including a short base width of 50 nm, moderate emitter and collector doping levels, and optimized GaN/β-Ga_2_O_3_ heterojunction alignment. Although β-Ga_2_O_3_ has inherently low electron mobility, the relatively thin base region and the high carrier saturation velocity in GaN help reduce the carrier transit time, thereby enhancing *f*_T_. Moreover, the combination of GaN and β-Ga_2_O_3_, two materials with wide bandgaps and high breakdown fields, enables robust high-frequency operation without premature breakdown. Compared to other wide-bandgap devices, the proposed β-Ga_2_O_3_/GaN HBT exhibits competitive high-frequency performance.^78,79^ The B-HBT achieves an *f*_T_ of 35 GHz, serving as an upper bound under bulk-trap-only conditions, while the F-HBT maintains an *f*_T_ of 30 GHz, comparable to reported values for β-Ga_2_O_3_ MOSFETs (27 GHz)^79^ and significantly exceeding that of AlGaN/GaN HBTs (4.05 GHz).^78^ Our simulations show that even under realistic full-trap conditions, the β-Ga_2_O_3_/GaN HBT sustains frequency performance on par with or better than established wide-bandgap technologies, while simultaneously providing superior breakdown capability. Furthermore, to the best of our knowledge, this study presents the first demonstration of a functional HBT based on the Ga_2_O_3_/GaN material system, even though Ga_2_O_3_/GaN heterojunctions have been extensively investigated for unipolar and optoelectronic devices such as p–n diodes, HEMTs, MOSFETs, and LEDs, as summarized in Table 1. By employing a p-GaN base to circumvent the long-standing challenge of p-type doping in β-Ga_2_O_3_, our work not only opens a new avenue for the development of Ga_2_O_3_-based bipolar devices but also contributes a novel device architecture to the wide-bandgap semiconductor field.

**Table 1:** Comparison of the current work with previously reported Ga_2_O_3_/GaN devices

Device	Key material layers	Primary function	Novelty	Reference
p–n diode	Ga_2_O_3_ (n-type), GaN (p-type)	High-performance rectifiers and power diodes with high breakdown voltage, low switching loss, and scalability for high-frequency power electronics	(i) β-Ga_2_O_3_/GaN Junction Barrier Schottky diode (JBSD) with high breakdown and fast switching; (ii) transfer-printed β-Ga_2_O_3_ nanomembranes for large-area devices; (iii) improved termination designs to maximize breakdown	44–47
HEMT	Ga_2_O_3_ layers integrated with GaN (heterojunction or gate oxide)	Fabrication of high-performance, normally-off, reliable HEMTs/MOSHEMTs for high-power and high-frequency applications	Using β-Ga_2_O_3_ as a gate dielectric/stack to enhance 2DEG density, raise threshold voltage, suppress leakage, and improve thermal/noise performance	25 and 32–38
MOSFET	β-Ga_2_O_3_ channel (grown by mist chemical vapor deposition (mist-CVD) on GaN substrates) and Ga_2_O_3_ gate dielectric (formed by photo-enhanced chemical (PEC) oxidation of GaN nanowires (NWs))	High-power, reliable, low-cost MOSFETs suitable for high-voltage power electronics	(i) PEC oxidation for Ga_2_O_3_/GaN hybrid NW MOSFETs; (ii) mist-CVD instead of MOCVD/MBE for cost-effective, high-performance β-Ga_2_O_3_ MOSFETs; (iii) very high breakdown voltage without complex structures	39–43
Photodetector	β-Ga_2_O_3_/GaN heterojunctions	Dual-mode, deep-UV high-selectivity, and high-resolution narrow-band UV photodetection	Bias-controlled dual-mode, dual-band high-responsivity, and ultranarrow spectral response	26–28
LED	GaN/InGaN LEDs on β-Ga_2_O_3_ substrates or with embedded patterned/nanorod Ga_2_O_3_ structures	High-efficiency GaN-based LEDs with improved light output, internal quantum efficiency, and photon extraction	First green LED on β-Ga_2_O_3_; Ga_2_O_3_ patterning and nanorods enhance light output power (LOP), internal quantum efficiency (IQE), and light extraction efficiency (LEE), while reducing strain and quantum-confined Stark effect (QCSE)	29–31
This work	n-β-Ga_2_O_3_ emitter/p-GaN base/n-GaN collector	A high-gain, high-frequency, high-breakdown transistor for next-generation power electronics and RF switching	First demonstration of a functional Ga_2_O_3_/GaN HBT *via* simulation, overcoming the lack of p-type β-Ga_2_O_3_, with realistic trap/interface modeling and structural optimization	

### 3.2 Temperature-dependent electrical characteristics of Ga_2_O_3_/GaN HBTs

For power electronics applications, particularly in power switching environments, the β-Ga_2_O_3_/GaN HBT must be capable of operating at elevated temperatures. As previously discussed, the combination of an ultra-wide bandgap β-Ga_2_O_3_ emitter with a high-performance p-GaN base and n-GaN collector provides a promising material platform, enabling the proposed β-Ga_2_O_3_/GaN HBT to achieve high performance. At room temperature, the B-HBT delivered a high collector current density (*J*_C_) of 26 kA cm^−2^ at a base current of 25 μA, corresponding to a power figure of merit (PFOM) of 74.8 MW cm^−2^ and a low specific on-resistance (*R*_on,sp_) of 0.19 mΩ cm^2^. In comparison, the F-HBT exhibited a *J*_C_ of 14.3 kA cm^−2^, yielding a PFOM of 41.3 MW cm^−2^ and a *R*_on,sp_ of 0.35 mΩ cm^2^. These results demonstrate strong competitiveness compared to conventional GaN-based HBTs.^76–78^ However, to assess the practical applicability of the device, it is essential to investigate the temperature dependence of key parameters such as collector current, current gain, specific on-resistance, and PFOM, in addition to performance under ideal conditions.

**Fig. 7:**
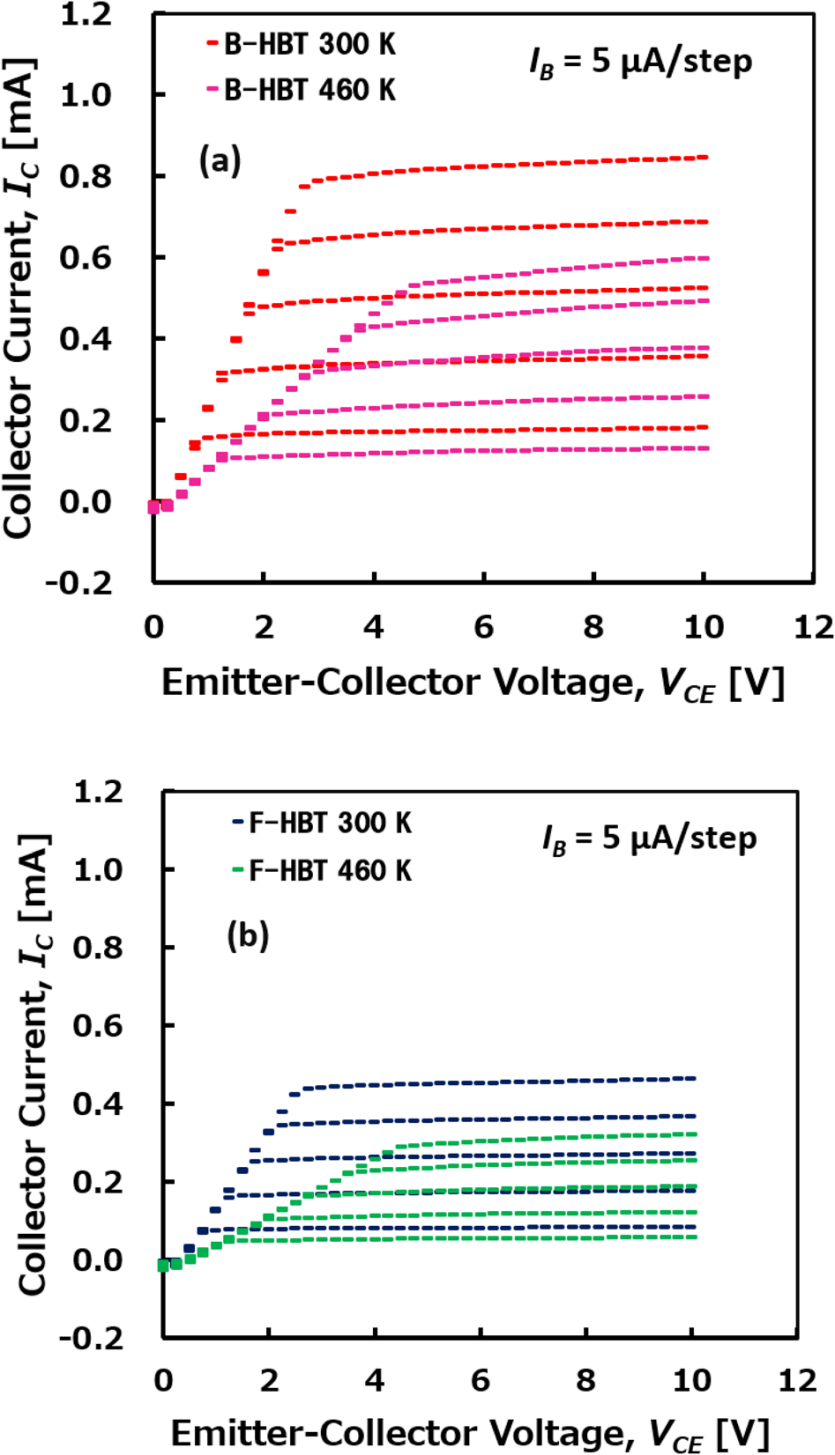
Common-emitter *I*–*V* characteristics of β-Ga_2_O_3_/GaN HBTs at 300 K and 460 K with a base-current step of 5 μA: (a) B-HBT (bulk-trap-only model) and (b) F-HBT (full-trap model).

**Fig. 8:**
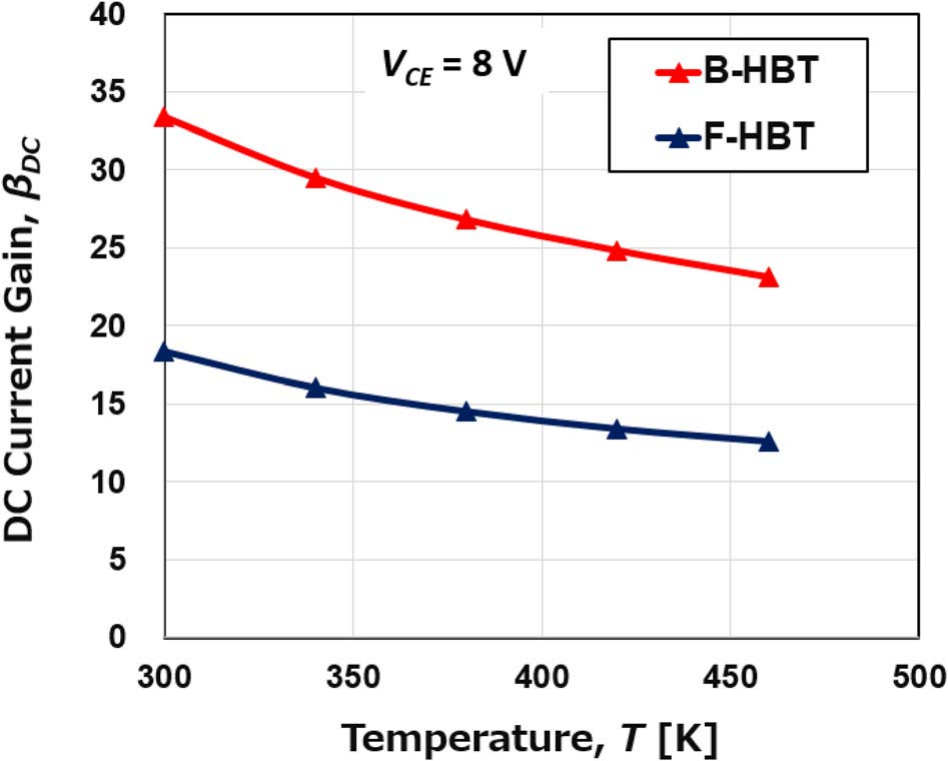
Temperature dependence of the DC current gain (*β*_DC_) of the B-HBT and F-HBT at a fixed base current of 25 μA.

**Fig. 9:**
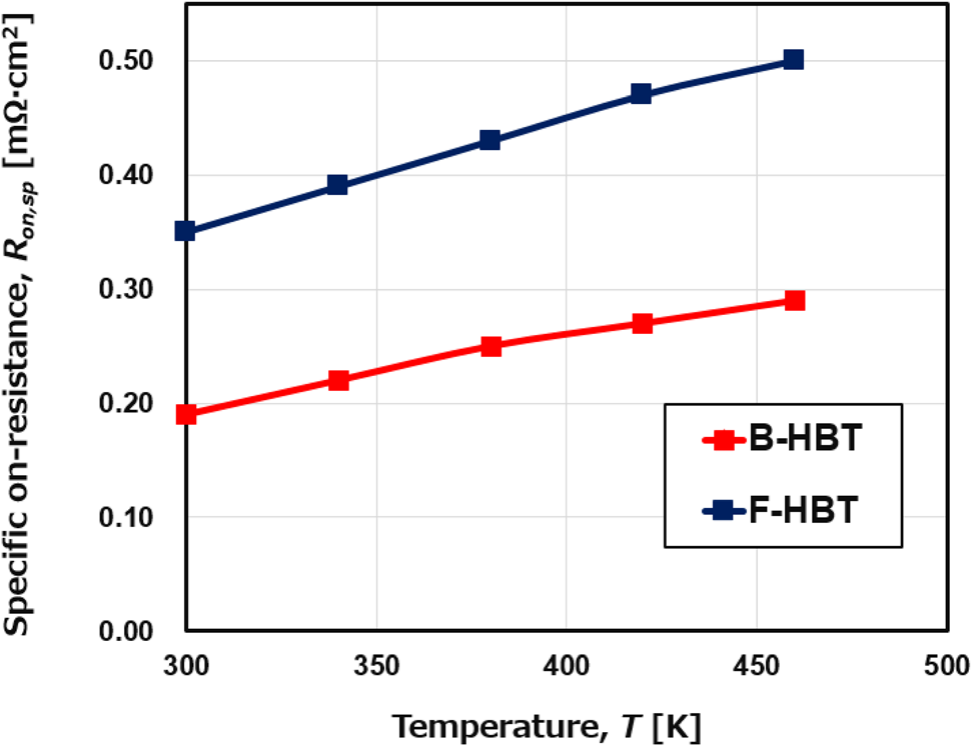
Temperature dependence of the specific on-resistance (*R*_on,sp_) of the B-HBT and F-HBT at a fixed base current of 25 μA.

**Fig. 10:**
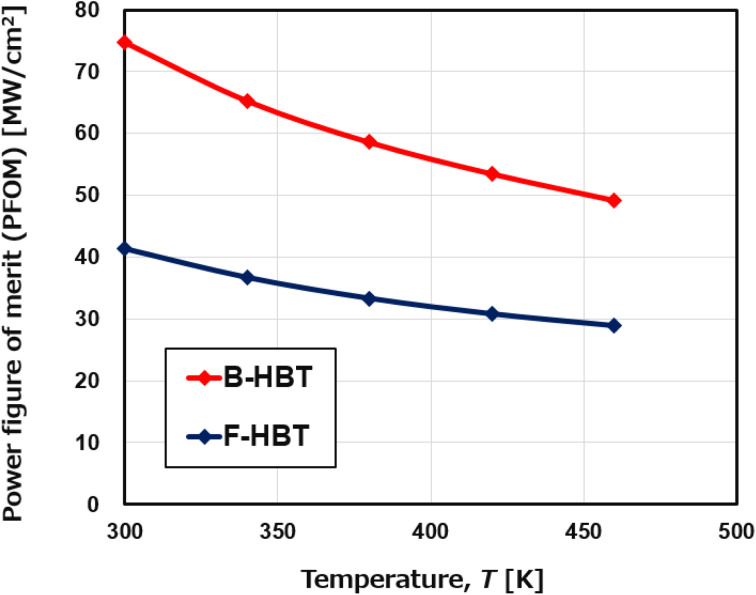
Temperature dependence of the power figure of merit (PFOM) of the B-HBT and F-HBT at a fixed base current of 25 μA.

**Fig. 11:**
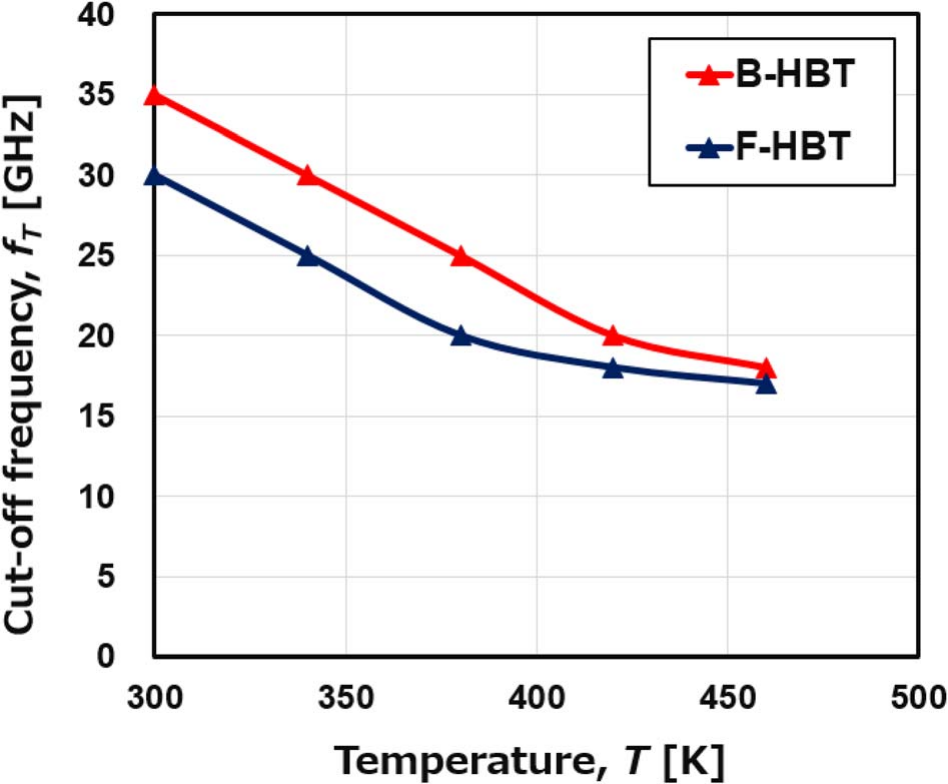
Temperature dependence of the cutoff frequency (*f*_T_) of the B-HBT and F-HBT.

The temperature-dependent performance of the Ga_2_O_3_/GaN HBT was systematically investigated over the range of 300 K to 460 K, as shown in Fig. 7–11. Fig. 7 compares the common-emitter *I*–*V* characteristics of the Ga_2_O_3_/GaN HBT at 300 K and 460 K with a base current step of 5 μA. A noticeable degradation in collector current is observed at 460 K compared to 300 K, especially at higher base current levels, indicating deteriorated carrier transport at elevated temperatures. However, at both temperatures, the *I*–*V* characteristics exhibit a distinct forward-active behavior, with a sharp increase in collector current (*I*_C_) at low *V*_CE_ followed by well-defined saturation regions. In addition, an increased offset voltage and reduced flatness in the saturation region at 460 K indicate mild base-width modulation and enhanced thermal effects. The B-HBT delivers higher *I*_C_ at both temperatures, whereas the F-HBT exhibits lower absolute *I*_C_ and poorer high-temperature performance, reflecting the impact of heterojunction-related non-idealities and compensation effects in the p-GaN base. Consequently, both devices exhibit reduced DC current gain (*β*_DC_) with temperature: the B-HBT decreases from 33.4 at 300 K to 23.1 at 460 K (31% reduction), while the F-HBT falls from 18.3 to 12.6 (31% reduction). Although the fractional degradation with temperature is similar, the F-HBT consistently operates with substantially lower absolute gain across the examined range, emphasizing the critical role of interface quality and defect control for reliable high-temperature Ga_2_O_3_/GaN HBT operation. The degradation in *β*_DC_ with increasing temperature is primarily attributed to enhanced carrier recombination in the base region and reduced carrier mobility at elevated temperatures, which together lower the emitter-to-base injection efficiency. In addition, higher temperatures facilitate hole back-injection across the emitter–base junction, further reducing injection efficiency. Moreover, the minority carrier lifetime decreases with temperature due to thermally activated deep-level traps and enhanced non-radiative recombination in both p-GaN and n-β-Ga_2_O_3_. The shortened lifetime accelerates recombination, decreases the base transport factor, and thereby further degrades the overall current gain. Compared with the B-HBT, the F-HBT suffers from stronger degradation because junction imperfections act as recombination centers and increase parasitic resistance, while compensating defects in the p-GaN base lower the effective hole concentration and weaken emitter injection efficiency.

Simultaneously, Fig. 9 and 10 show a consistent thermal response in both devices: as the temperature increases from 300 to 460 K, the specific on-resistance (*R*_on,sp_) rises monotonically, accompanied by a decline in the power figure of merit (PFOM). For the B-HBT, *R*_on,sp_ increases from 0.19 to 0.29 mΩ cm^2^ while PFOM drops from 74.8 to 49.2 MW cm^−2^. Likewise, the F-HBT shows a rise in *R*_on,sp_ from 0.35 to 0.50 mΩ cm^2^ and a reduction in PFOM from 41.3 to 28.9 MW cm^−2^. This degradation is attributed to thermally reduced electron mobility in the β-Ga_2_O_3_ emitter and drift regions. Importantly, the collector–base breakdown voltage (BV_CBO_) remains nearly constant at 120 V, confirming that the PFOM loss originates mainly from resistance increases rather than breakdown changes. Elevated resistance in the collector region also produces a larger voltage drop under fixed bias (*V*_C_), thereby reducing the effective junction voltage and lowering the collector current (*I*_C_). The combined effect of higher *R*_on,sp_ and suppressed *I*_C_ results in substantial PFOM degradation. This trend is corroborated by the output characteristics in Fig. 7, which show significantly higher *I*_C_ at 300 K compared to 460 K for the same collector–emitter voltage (*V*_CE_). The consistency between the *I*–*V* characteristics and PFOM decline highlights the thermal sensitivity of the device performance. This analysis demonstrates that although the proposed HBT structure maintains stable operation at elevated temperatures, both current gain and power efficiency are subject to significant thermal degradation, which must be carefully addressed in the design of high-temperature power and RF devices.

Heterojunction bipolar transistors (HBTs) are commonly used in practical RF and power switching applications, where they often operate under elevated thermal conditions. Therefore, evaluating the temperature dependence of the cutoff frequency (*f*_T_) is essential for understanding the high-frequency reliability and performance limits of the proposed Ga_2_O_3_/GaN HBT. As seen in Fig. 11, *f*_T_ decreases monotonically with temperature for both device variants. For the B-HBT, *f*_T_ falls from 35 to 18 GHz as *T* increases from 300 to 460 K, whereas the F-HBT decreases from 30 to 17 GHz. The full-trap device consistently exhibits lower *f*_T_ due to reduced transconductance and higher parasitic resistance/capacitance from interfacial defects; however, the gap between the two devices narrows at elevated temperatures where phonon-limited mobility dominates. This degradation is primarily driven by enhanced phonon scattering in the β-Ga_2_O_3_ emitter and GaN collector, which reduces carrier mobility, increases base transit time, and elevates diffusion capacitance and base resistance. Despite this, the proposed HBT maintains a competitive *f*_T_ across the investigated range. The wide bandgap of n-β-Ga_2_O_3_ ensures a high breakdown voltage, while the p-GaN base provides thermal stability along with efficient minority-carrier injection and fast switching. These combined attributes highlight the promise of β-Ga_2_O_3_/GaN HBTs for high-temperature, high-frequency power and RF applications.

### 3.3 Effect of base and collector thickness on the electrical performance of Ga_2_O_3_/GaN HBTs

The performance of heterojunction bipolar transistors (HBTs) can be enhanced by optimizing the base and collector thicknesses, specifically by balancing the trade-offs between current gain, breakdown voltage, and conduction loss. To clarify these effects, the electrical performance of the proposed β-Ga_2_O_3_/GaN HBT was evaluated as a function of base and collector thickness. First, all device parameters were kept constant while the base thickness (*t*_B_) was varied from 0.05 μm to 0.15 μm. For each *t*_B_, key parameters such as current gain, breakdown voltage, specific on-resistance (*R*_on,sp_), and power figure of merit (PFOM) were extracted and analyzed.

**Fig. 12:**
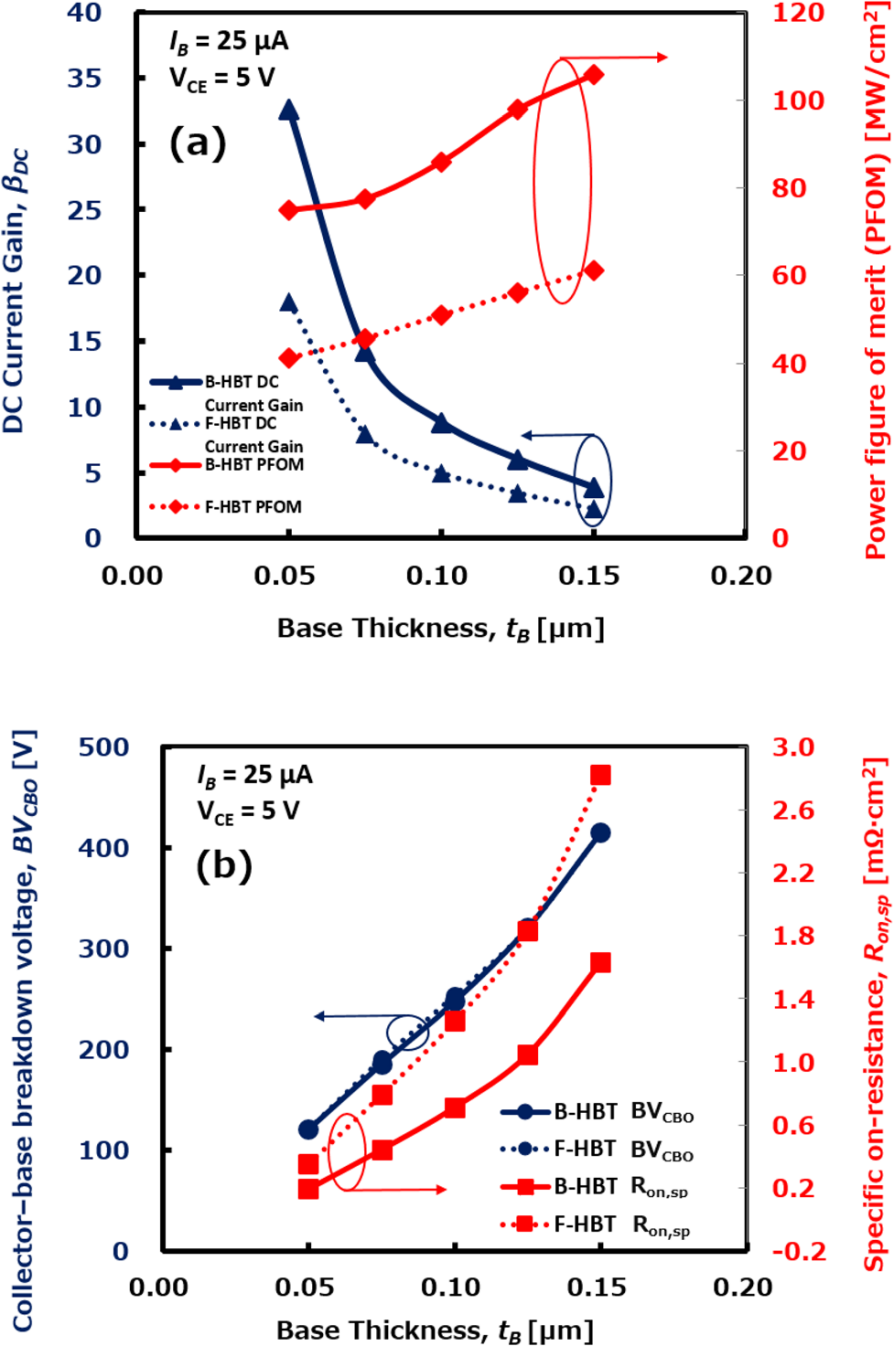
Dependence of (a) DC current gain (*β*_DC_) and power figure of merit (PFOM), and (b) collector–base breakdown voltage (BV_CBO_) and specific on-resistance (*R*_on,sp_) on base thickness (*t*_B_) for the B-HBT and F-HBT, evaluated at *I*_B_ = 25 μA and *V*_CE_ = 5 V.

Fig. 12(a) presents the dependence of DC current gain (*β*_DC_) and power figure of merit (PFOM) on base thickness (*t*_B_). As *t*_B_ increases from 0.05 μm to 0.15 μm, *β*_DC_ for both the B-HBT and F-HBT decreases significantly due to enhanced carrier recombination and prolonged transit time through the thicker base region. In contrast, PFOM generally increases with *t*_B_, primarily driven by the substantial enhancement in breakdown voltage, which outweighs the concurrent increase in specific on-resistance (*R*_on,sp_), as shown in Fig. 12(b). Across the entire thickness range, the F-HBT exhibits lower *β*_DC_ and PFOM than the B-HBT, consistent with additional interfacial traps and parasitic resistances at the β-Ga_2_O_3_/GaN junction that hinder carrier transport. As seen in Fig. 12(b), the breakdown voltage of both devices increases markedly with *t*_B_, since a thicker base broadens the depletion region, thereby improving electric field distribution and enhancing voltage blocking capability. Meanwhile, *R*_on,sp_ exhibits a noticeable increase with *t*_B_ due to the longer transport path and higher series resistance in the base/collector stack. Adjusting the base thickness proves to be an effective strategy for balancing switching efficiency and voltage robustness in Ga_2_O_3_/GaN HBTs. The base thickness (*t*_B_) critically influences the overall device performance. A thinner base significantly enhances the DC current gain (*β*_DC_) by reducing carrier transit time and minimizing recombination within the base region, which are essential factors for high-gain amplification and RF operation. However, a thin base limits the breakdown voltage and PFOM due to a narrower depletion region and weaker electric field control. In contrast, increasing the base thickness substantially improves breakdown voltage and PFOM, although it leads to a degradation in *β*_DC_. Therefore, an optimized base thickness offers a favorable trade-off. It provides sufficient current gain for efficient switching while maintaining high power performance and voltage robustness, which are critical for the practical implementation of Ga_2_O_3_/GaN HBTs in high-voltage and high-frequency power applications.

**Fig. 13:**
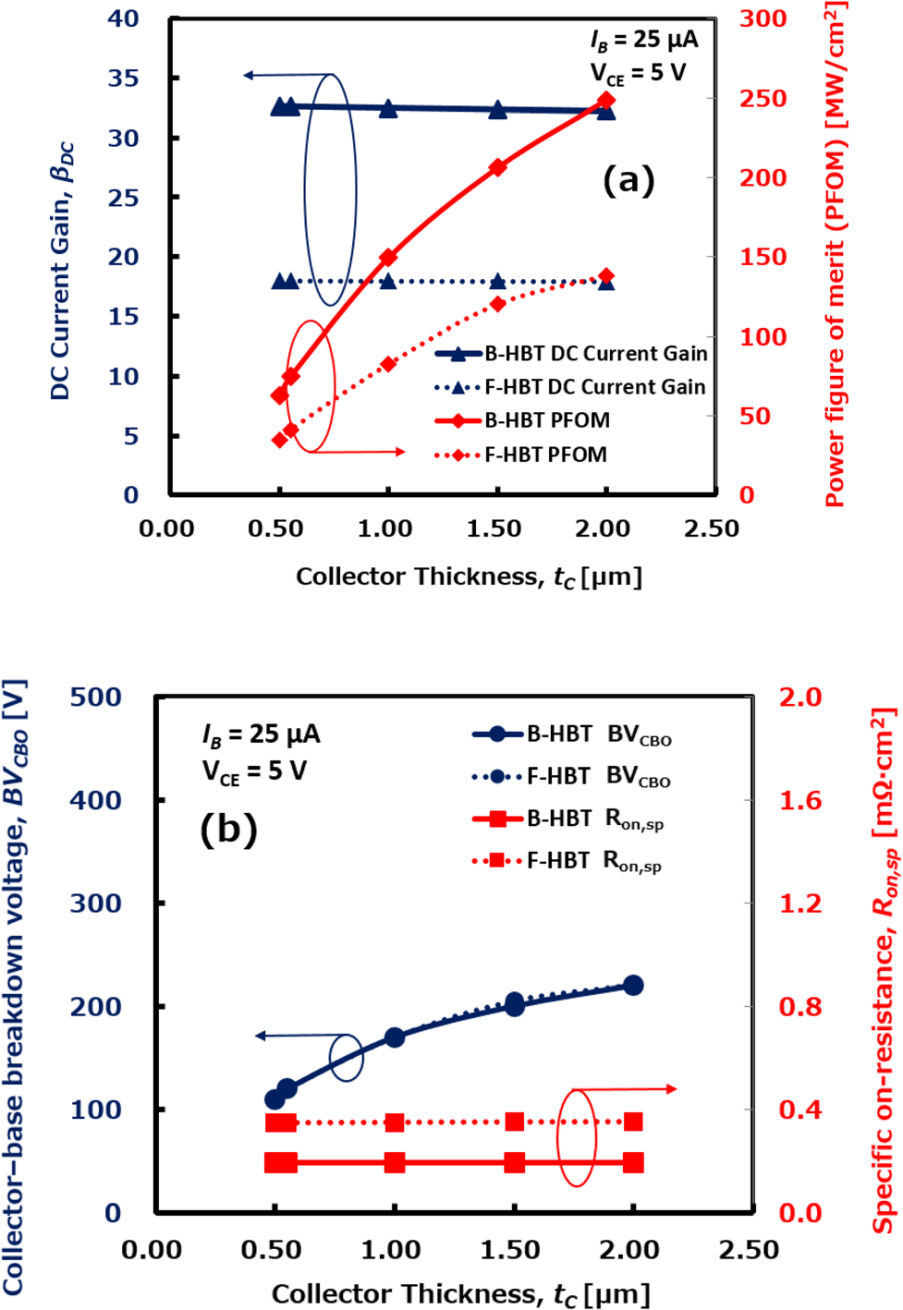
Dependence of (a) DC current gain (*β*_DC_) and power figure of merit (PFOM), and (b) collector–base breakdown voltage (BV_CBO_) and specific on-resistance (*R*_on,sp_) on collector thickness (*t*_C_) for the B-HBT and F-HBT, evaluated at *I*_B_ = 25 μA and *V*_CE_ = 5 V.

Next, the effect of collector thickness (*t*_C_) on device performance was investigated by varying *t*_C_ from 0.5 μm to 2.0 μm while keeping all other HBT parameters constant. The dependence of key performance metrics on collector thickness was systematically analyzed, and the corresponding simulation results are presented in Fig. 13. As illustrated in Fig. 13(a), the DC current gain (*β*_DC_) of both B-HBT and F-HBT remains nearly constant, at approximately 32 for the B-HBT and 18 for the F-HBT, across the entire range of collector thickness (*t*_C_). This suggests that increasing the collector thickness does not significantly influence carrier injection efficiency or recombination within the base, as *β*_DC_ is primarily determined by the emitter–base junction characteristics. In contrast, the power figure of merit (PFOM) exhibits a substantial enhancement, rising from 62.9 MW cm^−2^ at *t*_C_ = 0.5 μm to over 248 MW cm^−2^ at *t*_C_ = 2.0 μm for the B-HBT, and from 34.7 MW cm^−2^ to over 138 MW cm^−2^ for the F-HBT over the same thickness range. This improvement is mainly due to the increase in breakdown voltage (BV_CBO_) with thicker collector layers, as shown in Fig. 13(b). A thicker collector enables a wider depletion region and a more uniform electric field distribution, thereby enabling significantly higher breakdown voltage. Specifically, BV_CBO_ increases from approximately 110 V to 220 V for both devices as *t*_C_ increases. Notably, the specific on-resistance (*R*_on,sp_) shows only minor variation across the same thickness range, indicating that enhanced thickness does not introduce significant resistance penalties. Although both devices benefit from collector thickening, the F-HBT consistently exhibits a lower DC current gain, which can be attributed to defect-related recombination at the Ga_2_O_3_/GaN junction arising from interfacial imperfections and structural mismatches. Consequently, the F-HBT also demonstrates a lower PFOM across the entire range, since such interfacial defects raise parasitic resistances. These results demonstrate that optimizing the collector thickness is an effective strategy for enhancing the breakdown strength and power efficiency of Ga_2_O_3_/GaN HBTs, without compromising DC current gain or *R*_on,sp_. Furthermore, this approach offers a promising solution for improving the trade-off between high-voltage capability and conduction loss in high-power switching applications.

Our simulations show that both base thickness (*t*_B_) and collector thickness (*t*_C_) play pivotal roles in determining the overall performance of Ga_2_O_3_/GaN HBTs, although their effects manifest in distinct ways. A thinner base is favorable for applications requiring high gain and fast response, but it compromises breakdown voltage and PFOM. In contrast, increasing the collector thickness exhibits a dominant and beneficial impact on key performance metrics; a thicker collector enhances both breakdown voltage and PFOM without causing significant degradation in *β*_DC_ or *R*_on,sp_. This finding is scientifically significant because it reveals a practical and fabrication-compatible approach to improve voltage endurance and switching efficiency without sacrificing current gain. This trade-off between current gain and breakdown performance has long been a challenge in HBT design. Therefore, a structural configuration with a thin base and a moderately thick collector is recommended to achieve optimized performance. This combination supports a favorable balance among DC current gain, power efficiency, and voltage robustness, making it particularly suitable for high-voltage, high-frequency power switching, compact power ICs, and RF front-end modules.

Table 2 provides a comparative overview of the key performance metrics of the proposed Ga_2_O_3_/GaN HBT in relation to previously reported wide-bandgap devices. As shown in Table 2, experimental AlGaN/GaN HBTs^78^ exhibit very high current gain (up to 129) but suffer from limited cutoff frequencies (<5 GHz), whereas InGaN/GaN HBTs^77,81^ deliver moderate current gain but relatively low breakdown voltages (∼95–105 V). Simulation results of Cu_2_O/β-Ga_2_O_3_ HBTs^23^ indicate the potential for high breakdown voltages (>3 kV); however, the narrow bandgap of the p-type base material fundamentally limits their performance. In contrast, the simulated Ga_2_O_3_/GaN HBT proposed in this work achieves a well-balanced trade-off among current gain, breakdown voltage, PFOM, and frequency response, thereby positioning it as a promising candidate for next-generation power and RF electronic applications. By comparing the characteristics of the B-HBT and F-HBT, it becomes evident that the Ga_2_O_3_/GaN heterointerface is the primary bottleneck for achieving high-performance HBTs. Our simulations further reveal that only a low density of interface trap (*D*_it_ ≈ 1.0 × 10^12^ eV^−1^ cm^−2^) is compatible with transistor operation, whereas the higher trap densities reported experimentally (2.3–5.3 × 10^13^ eV^−1^ cm^−2^) would completely suppress device functionality. The feasibility of Ga_2_O_3_/GaN HBTs therefore hinges on improving interface quality through optimized growth processes, suppression of oxygen diffusion, and advanced strain engineering. It is concluded that stringent interface control is essential as the critical pathway to translate the promising theoretical performance of Ga_2_O_3_/GaN HBTs into practical applications.

**Table 2:** Comparison of the proposed Ga_2_O_3_/GaN HBT with previously reported wide-bandgap devices

Device structure	DC current gain (*β*_DC_)	Breakdown voltage (V)	*R*_on,sp_ (mΩ cm^2^)	PFOM (MW cm^−2^)	*f*_T_ (GHz)	Year	Reference
Ga_2_O_3_/GaN HBT (B-HBT)	34.9	120	0.19	74.8	35	2025	This work
Ga_2_O_3_/GaN HBT (F-HBT)	18.3	120	0.35	41.3	30	2025	This work
Cu_2_O/β-Ga_2_O_3_ HBT	>50	3540–280	—	34.0	—	2023	23
AlGaN/GaN HBT	129	160	0.28	91.0	4.05	2022	78
AlGaN/GaN HBT	25	—	—	—	—	2022	72
AlGaN/GaN HBT	2	—	—	—	—	2016	80
InGaN/GaN HBT	24	105	0.14	∼78.7	—	2013	81
GaN/InGaN HBT	>24	>95	—	—	> 5	2011	77
AlGaN/GaN HBT	18	>330	—	—	—	2003	73
GaN/AlGaN HBT	10	—	—	—	—	2000	71
β-Ga_2_O_3_ MESFET	—	150	—	—	27	2019	79

## 4 Conclusions

In this work, a β-Ga_2_O_3_/GaN heterojunction bipolar transistor (HBT) featuring an n-type β-Ga_2_O_3_ emitter, a p-type GaN base, and an n-type GaN collector was designed and simulated to overcome the limitations of β-Ga_2_O_3_ bipolar devices caused by the absence of reliable p-type doping. Our simulation results confirm, for the first time, that the β-Ga_2_O_3_/GaN structure can effectively function as a heterojunction bipolar transistor. The proposed device with full consideration of traps achieves a high DC current gain of 18.3 and an ultra-low specific on-resistance (*R*_on,sp_) of 0.35 mΩ cm^2^, resulting from efficient carrier injection across the n-β-Ga_2_O_3_/p-GaN heterojunction and optimized layer thicknesses. The collector–base breakdown voltage (BV_CBO_) reaches 120 V, yielding a power figure of merit (PFOM) of 41.3 MW cm^−2^. A cutoff frequency (*f*_T_) of 30 GHz demonstrates competitive high-frequency performance. Moreover, the device retains stable operation at elevated temperatures up to 460 K, despite predictable degradation in DC current gain and PFOM, primarily due to reduced carrier mobility and increased recombination. Structural optimization further shows that a thin base preserves high gain, while a thicker collector improves PFOM by enhancing breakdown voltage without degrading *R*_on,sp_ or *β*_DC_. These findings highlight the strong potential of the Ga_2_O_3_/GaN HBT for high-voltage and high-frequency applications, positioning it as a promising candidate to bridge the performance gap between ultra-wide-bandgap oxides and III-nitride semiconductors.

## Author contributions

Phuc Hong Than: conceptualization, formal analysis, investigation, methodology, resources, validation, writing – original draft, writing – review & editing; Tho Quang Than: conceptualization, data curation, formal analysis, investigation, methodology, writing – review & editing; and Yasushi Takaki: conceptualization, investigation, formal analysis, resources, writing – review & editing.

## Conflicts of interest

The authors declare no conflict of interest.

## Data Availability

The data that support the findings of this study are available from the corresponding author upon reasonable request.
